# A Survey on Secure WiFi Sensing Technology: Attacks and Defenses

**DOI:** 10.3390/s25061913

**Published:** 2025-03-19

**Authors:** Xingyu Liu, Xin Meng, Hancong Duan, Ze Hu, Min Wang

**Affiliations:** 1 School of Electrical Information, Southwest Petroleum University, Chengdu 610500, China; 202222000120@stu.swpu.edu.cn (X.L.);; 2 School of Computer, University of Electronic Science and Technology of China, Chengdu 611731, China

**Keywords:** WiFi sensing, wireless security, WiFi localization, privacy-preserving

## Abstract

As a key enabling technology of the Internet of Thing (IoT), WiFi sensing has undergone noteworthy advancements and brought significant improvement to prevailing IoT systems and applications. The past few years have witnessed growing efforts in WiFi sensing, which is widely applied in various applications, such as indoor localization, human activity recognition, physiological signal monitoring, and so on. However, these techniques are also maliciously used by attackers to eavesdrop on legitimate users and even tamper the sensing results. Fortunately, these attack techniques in turn promote the advancement of WiFi sensing techniques, especially defense techniques. In this study, we carried out a comprehensive survey to systematically summarize the works related to the topic of attacks and defenses on WiFi sensing technology. Firstly, we summarize the existing surveys in related areas and highlight our unique novelty. Then, we introduce the concept of the core topic of this survey and provide a taxonomy to distinguish different kinds of attack and defense techniques, respectively, that is, active and passive attack techniques as well as active and passive defense techniques. Furthermore, existing works in each category are grouped and introduced in detail, respectively.

## 1. Introduction

Over the decades, IoT-related technologies have gone through rapid development [1,2,3,4,5,6,7,8,9], and the physical world and the information world are now tightly connected. In particular, wireless sensing, one of the key enabling technologies of IoT, revolutionizes the traditional sensing paradigm with the advantages of its non-intrusiveness, ubiquity, and ease of deployment with wireless sensors and signals [10,11,12,13,14]. This flexibility makes wireless sensing an invaluable tool in various applications, such as health monitoring [15,16,17], location-based services [18,19,20,21], intelligent transportation [22,23,24], and so on [25,26,27].

Existing efforts in wireless sensing exploit diverse wireless signals, such as radio-frequency identification (RFID) [28,29,30,31,32], acoustic [25,33,34,35,36,37], WiFi [15,16,17,38], mmWave [18,26,39,40,41,42,43] signals, etc. Among these different sensing technologies, WiFi-based technologies are the most commonly used because WiFi networks are already widespread in homes, offices, and public spaces, making WiFi sensing highly accessible. Its widespread availability eliminates the need to deploy additional sensing infrastructure, significantly reducing costs. Most current WiFi-based sensing methods perform sensing tasks either by exploiting the received signal strength indicator (RSSI) to measure and analyze the power level of the received WiFi signal [44] or by using channel state information (CSI) to find fine-grained information in the amplitude and phase of subcarrier signals [7,45,46,47]. Moreover, recent advancements in WiFi-based integrated sensing and communication (ISAC) [48,49] have significantly enhanced the potential for future applications of WiFi sensing. These developments can enable more sophisticated and accurate sensing capabilities by combining the strengths of both WiFi-based sensing and communication technologies.

However, the ease of access and ubiquity of WiFi signals and access points (APs) have raised significant security concerns and threats [50,51,52]. WiFi networks are pervasive in both public and private spaces, making them an attractive target for malicious attackers. The sophisticated nature of WiFi sensing technologies, which allows for detailed environmental and user data collection, exacerbates these concerns. Unlike traditional WiFi usage, which primarily focuses on data transmission, WiFi sensing can capture sensitive information such as user movements, locations, and even breathing patterns [15,16,17,53]. This capability, while beneficial for legitimate applications, opens the door to various security vulnerabilities.

There are two main security threats identified by existing works targeting WiFi sensing. One method is the active attack, where attackers can access WiFi networks from a distance to tamper with WiFi devices and signals, thereby manipulating the sensing results [51,54]. Another method is the passive attack, where attackers receive and analyze WiFi signals to eavesdrop on legitimate users’ private sensing results, such as tracking when a person is home and monitoring their daily routines [50,55]. In response to these security threats, several effective countermeasures have been proposed to protect legitimate users and sensing targets. On one hand, some methods adopt active defense, where users manipulate WiFi signals to mislead attackers, causing them to obtain incorrect sensing results [56,57]. On the other hand, some other methods perform passive defense, where a third party protects the users’ sensing results or alerts users to the risk of their sensing data being leaked [58,59].

Although it is possible to create a more secure environment for WiFi sensing by employing existing defense strategies, secure WiFi sensing still faces unique technical challenges in preserving WiFi communication links and being transparent to legitimate users. Even though existing works have attempted to overcome these challenges by designing novel sensing techniques, these works are still removed from ubiquitous and practical deployment. Thus, it is important to figure out the challenges they face and the limitations that have not been addressed. Moreover, the development trends should be pointed out to illuminate the potential directions of secure WiFi sensing. Therefore, there is a strong need for a comprehensive study of secure WiFi sensing.

To this end, we conducted a systematic survey of secure WiFi sensing technology, including both the methods to attack WiFi sensing systems and the methods to defend against the corresponding attacks. As for the attack methods, in most cases, attackers either actively tamper with the sensing results or passively eavesdrop on the sensing data, so we categorized the existing attack methods into active attack and passive attack. As for the defense methods, in most cases, users either actively adopt defense strategies themselves or are passively secured by a third party, so we also categorized the existing defense methods into active defense and passive defense. Research works in each category were well discussed and compared, respectively.

There have been some related surveys that provide a summary of particular scopes, such as secure wireless communication, IoT security, and WiFi sensing. We compared and herein summarize them in detail, finding that none of the current surveys focus on summarizing the existing technologies for attack on and defense of WiFi sensing and analyzing their key challenges. As we mentioned, a comprehensive survey is necessary and important for researchers and application developers. This survey is expected to inspire researchers and developers to conduct further research in secure WiFi sensing and build various applications to realize privacy-preserving, reliable, ubiquitous, and accurate WiFi sensing in real life.

The main contributions of this paper are summarized as follows:

- **Complete picture and novel taxonomy:** We present a complete picture of the literature in the area of secure WiFi sensing and propose a novel taxonomy for technologies for attack and defense of WiFi sensing, respectively, which guides the first step;
- **Inclusive and up-to-date coverage:** Our survey is inclusive, encompassing the latest advancements in secure WiFi sensing technologies. By integrating recent developments, we ensure that our work remains relevant and valuable to the research community;
- **Comprehensive comparison and summary:** We comprehensively compare and summarize existing technologies for attack and defense of WiFi sensing to provide readers with a thorough understanding of this area;
- **Identification of key challenges and future directions:** Our survey discusses the key challenges and future directions, which may inspire researchers and developers to carry on further research in WiFi sensing and build various applications to realize privacy-preserving, reliable, ubiquitous, and accurate WiFi sensing.

**Figure 1:**
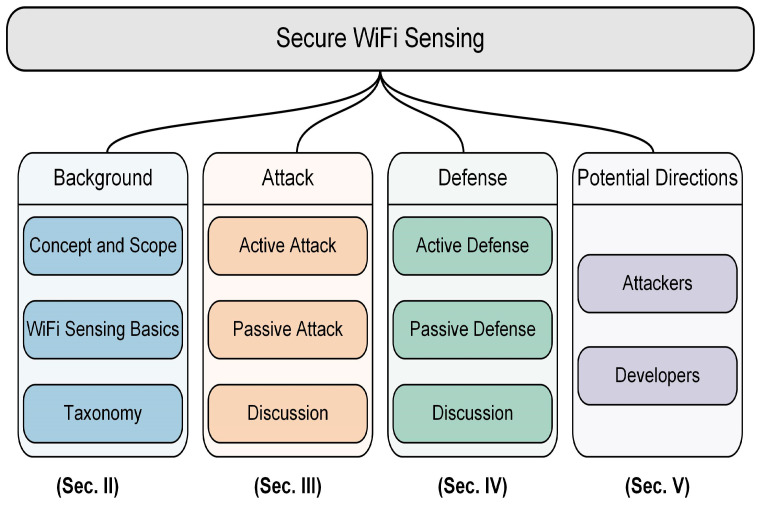
Outline of this survey.

As shown in Figure 1, the structure of this survey is organized as follows. Section 2 introduces the scope and taxonomy of this survey. Section 3 and Section 4, respectively, summarize and discuss the research works of attacks against and defenses on existing WiFi sensing systems. Based on the analysis and summary of these works, Section 5 discusses potential challenges and opportunities on the topic of secure WiFi sensing. Finally, we conclude this survey in Section 6.

## 2. Background

In this section, we introduce the background of this survey. We first present the concept of secure WiFi sensing and clarify the scope of this survey. Following that, we briefly introduce the basic metric enabling WiFi sensing, WiFi CSI. Then, we explain our taxonomy of the existing works as well as the definition of each category.

### 2.1. Concept and Scope

Secure WiFi sensing is a technology that uses WiFi signals to perform sensing tasks (e.g., location estimation, health monitoring, and activity recognition) while ensuring data privacy and security. In the traditional WiFi sensing process, WiFi signals are emitted by the transmitter (Tx) and then propagate through the wireless channel so that the signal properties (e.g., amplitude, phase, frequency, and polarization) are changed due to the existence or movement of sensing targets. A WiFi receiver (Rx) can receive these WiFi signals and employ different sensing algorithms to extract the sensing information of the sensing target. However, there might be malicious attackers who try to attack the WiFi sensing systems, for example, by means of jamming or eavesdropping. Secure WiFi sensing aims to explore adequate security strategies to defend against these attacks, protecting user privacy and ensuring reliable data interpretation.

The scope of this survey contains the attack techniques and defense strategies related to WiFi sensing technology.

### 2.2. WiFi Sensing Basics

In this section, the basic metric enabling WiFi sensing is WiFi CSI. CSI refers to the detailed data that describe how a WiFi signal propagates from the transmitter to the receiver, including how the signal is affected by obstacles, reflections, and scattering along the path [44]. Essentially, CSI provides a fine-grained representation of the wireless channel at the level of individual subcarriers, which are components of the overall WiFi signal. WiFi networks, particularly those using the IEEE 802.11n/ac/ax standards, employ Orthogonal Frequency-Division Multiplexing (OFDM). OFDM divides the WiFi channel into multiple subcarriers, each carrying a portion of the data. CSI captures the amplitude and phase information for each subcarrier, providing a snapshot of the wireless channel at a given moment.

The CSI for a WiFi signal is often represented as a complex matrix *H*, where each element corresponds to the channel response of a specific subcarrier between a Tx–Rx antenna pair. The matrix H can be expressed as follows:

$$H=\left[\begin{array}{ccc} h_{11} & \cdots & h_{1N}\\ \vdots & \ddots & \vdots \\ h_{M1} & \cdots & h_{MN} \end{array}\right]$$

where M is the number of transmit antennas, N is the number of receive antennas, and $h_{MN}$ represents the channel response of the *m*th transmit antenna to the *n*th receive antenna for a specific subcarrier.

Specifically, for a subcarrier with center frequency *f*, the corresponding channel response is as follows:

$$h=\sum a\left(t\right)e^{-j\phi \left(t\right)}$$

where $a\left(t\right)$ is the amplitude attenuation factor and phase, and $\phi \left(t\right)$ is the phase shift, respectively.

The CSI amplitude and phase are impacted by the displacements and movements of the transmitter, receiver, and surrounding objects and humans. In other words, CSI captures the wireless characteristics of the nearby environment. These characteristics, assisted by mathematical modeling or machine learning algorithms, can be used for different sensing applications. This is the rationale for why CSI can be used for WiFi sensing.

### 2.3. Taxonomy

Research works on the topic of secure WiFi sensing can be categorized into a two-level structure. The methods are first categorized by their purposes: attack or defense.

For the attack techniques against WiFi sensing, they are further divided into two categories according to whether the attacker interferes with the sensing system.

- **Active attack.** (1) Definition: Attacks that involve direct interaction with the WiFi sensing system to alter, disrupt, or damage the wireless channel or its operations. (2) Objective: The attacker aims to interfere with the normal functioning of the sensing system, causing it to produce inaccurate or unusable results;
- **Passive attack.** (1) Definition: Attacks that focus on monitoring or eavesdropping on the wireless channel or sensing results without altering them. (2) Objective: The attacker aims to gather sensitive information or infer private data without being detected.

The defense techniques for WiFi sensing are also divided into two categories according to whether the defense strategies are adopted by the sensing target.

- **Active defense.** (1) Definition: Protective measures taken directly by the sensing target (e.g., a user, device, or system) to safeguard against attacks. (2) Objective: The target actively engages in its own protection to mitigate potential threats;
- **Passive defense.** (1) Definition: Protective measures adopted by external parties, such as users of the sensing data or third-party entities, to secure the sensing information. (2) Objective: These measures protect the sensing data or system without requiring direct involvement from the sensing target.

## 3. Attacks Against WiFi Sensing

In this section, we elaborate on the attack techniques against WiFi sensing. As summarized in Table 1, we first introduce the related works on active and passive attacks, respectively.

### 3.1. Active Attack

Active attacks mainly focus on direct interaction with the WiFi sensing system to alter, disrupt, or damage the wireless channel. The attacker aims to interfere with the normal operation of the sensing system by manipulating signal transmissions or the communication environment [51,54,60,61,62,63,64,65]. This interference can degrade the sensing performance or compromise data integrity to intentionally introduce anomalies or distortions in the wireless signals, affecting the system’s ability to accurately interpret environmental information. By directly targeting the signal propagation or transmission processes, these attacks pose significant challenges to the reliability and security of WiFi sensing applications.

**Figure 2:**
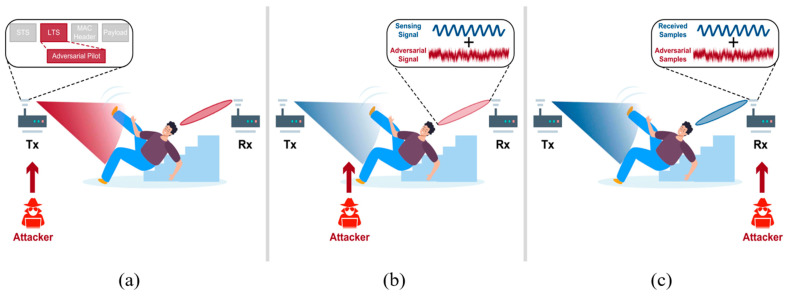
Illustration of attacks on different objects in WiFi recognition systems (e.g., Tx, channel, and Rx): (**a**) Tx [51]; (**b**) channel [60]; (**c**) Rx [64].

The mainstream of active attack techniques is attacking WiFi recognition systems, e.g., gesture recognition and activity recognition. The WiFi signal is emitted by Tx, is then propagated through the wireless channel, and is finally received by Rx. Therefore, there are works that attack these three different objects, as illustrated in Figure 2.

Li et al. [51] demonstrated how adversaries can attack Tx to subtly manipulate WiFi packet preambles to affect CSI and thereby influence underlying deep learning models (shown in Figure 2a). With the analysis of the relationship between the pilot symbol and CSI, a delicate mechanism is proposed to facilitate quantitative control of receiver-side CSI through minimal modifications to the pilot symbols of WiFi packets at the Tx. Moreover, by using a perturbation optimization method based on the Carlini and Wagner (CW) attack and a penalty-based training process, this attack method can be universally effective across various CSI responses and noise.

The attacks on Rx mainly try to manipulate the samples of training datasets or inject adversarial samples into the benign datasets, which is illustrated in Figure 2c. WiCAM [64] uses an adversarial perturbation added to the CSI of the received WiFi signal, which minimizes the influence on communication while retaining the attack effect. WiCAM utilizes an attention-based framework to identify the CSI subcarriers important to sensing, only adding limited perturbations to these critical parts. Then, the added perturbations have the strongest adversarial effect but incur the minimum influence on bit error rate (BER). Song et al. [62] investigated the effectiveness of attacking WiFi sensing systems via false data injection (FDI). They perform different types of FDI in seven datasets to reveal the decline in sensing accuracy.

**Figure 3:**
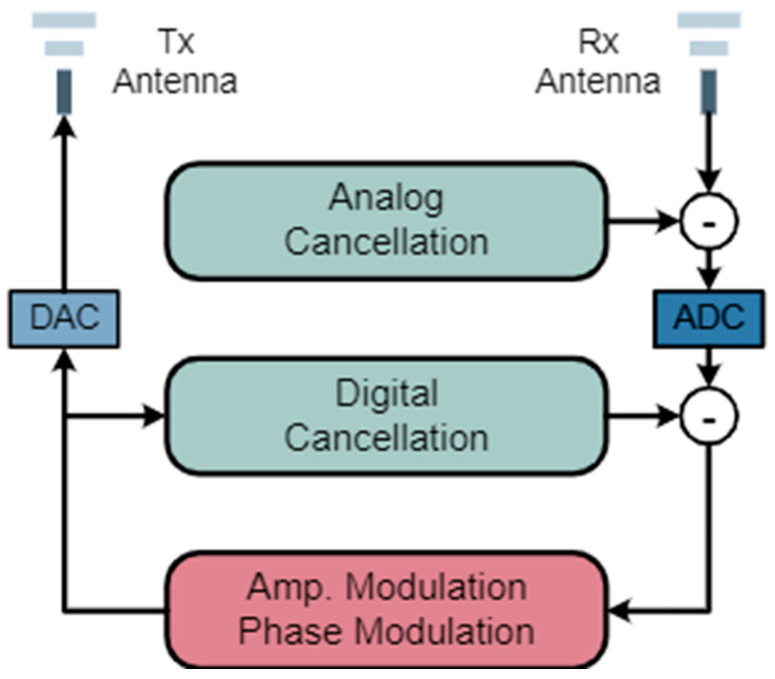
The design of the full-duplex attacker in WiAdv [60].

Besides attacking Tx or Rx, most of the works try to attack the wireless channel because this open medium is the easiest to invade and manipulate. Liu et al. [61] exploited the phenomenon of CSI absence in WiFi communication to affect the received WiFi signals. Therefore, by carefully designing the pattern of CSI absence, the sensing results can be modified purposefully. However, this attack may degrade the communication quality (e.g., packet loss and signal-to-noise ratio), thus making the attacks noticeable to users. To keep the stealthiness of the attack, WiAdv [60] features a signal synthesis scheme to craft adversarial WiFi signals without affecting the communication quality. WiAdv exploits full-duplex devices (as Figure 3 shows) to tactfully forward WiFi signals to mimic dynamic multipath, which makes the artificial multipath indistinguishable from the natural ones from the perspective of WiFi Rx.

Different from many methods assuming a clean wireless environment, the use of IS-WARS [54] demonstrated that there is widely existing interference from coexisting protocols such as ZigBee and Bluetooth. These signals can also be used to attack WiFi sensing systems to compromise the recognition process. Thus, IS-WARS uncovers new adversarial attacks against WiFi activity recognition systems by intentionally injecting interference using coexisting protocol signals. This adversarial attack is stealthy in terms of avoiding being detected by traffic analysis.

In recent years, metasurface has been widely used to manipulate wireless signals and program wireless environments [68,69,70,71,72,73,74]; thus, there are some attempts being made to use metasurface for WiFi attacks. RIStealth [65] leverages a delicate metasurface to render a moving individual undetectable by WiFi-based intrusion detection systems. By using the designed metasurface to perform fine-grained manipulation of the reflected WiFi signal, an intruder can approach the protected area sneakily and lift the threshold of the alert based on the basic principle of most intrusion detection systems. Thus, the intruder can make himself invisible.

### 3.2. Passive Attack

Passive attacks primarily focus on monitoring or eavesdropping on the wireless channel and the sensing results without altering or interfering with them [52,55,66,67]. In these attacks, the attacker remains undetected while collecting valuable information from the system. The objective is to silently gather data on the WiFi sensing environment, such as user movements, device activity, or other sensitive information, without directly interacting with or affecting the system’s operations. These attacks are particularly concerning in scenarios where sensitive data are being transmitted or where long-term monitoring can reveal patterns and behaviors without the knowledge of the system’s users.

Shi et al. [66] conducted a passive attack on public WiFi sensing datasets and investigated the leakage of private attributes. They found that public WiFi sensing datasets (e.g., gesture recognition dataset WiDar [75] and activity recognition dataset WiAR [76]) will leak users’ private attributes (e.g., height, weight, and gender) when attacked by a specially designed deep learning model.

Hernandez et al. [55] studied the feasibility of monitoring the victims via through-wall WiFi sensing. They demonstrated that with a proper analysis of CSI collected by the behind-wall attacker, the attacker can recognize both the presence of targets as well as their moving direction in a room. This private information may be leveraged to track and count the flow of traffic throughout a building.

**Figure 4:**
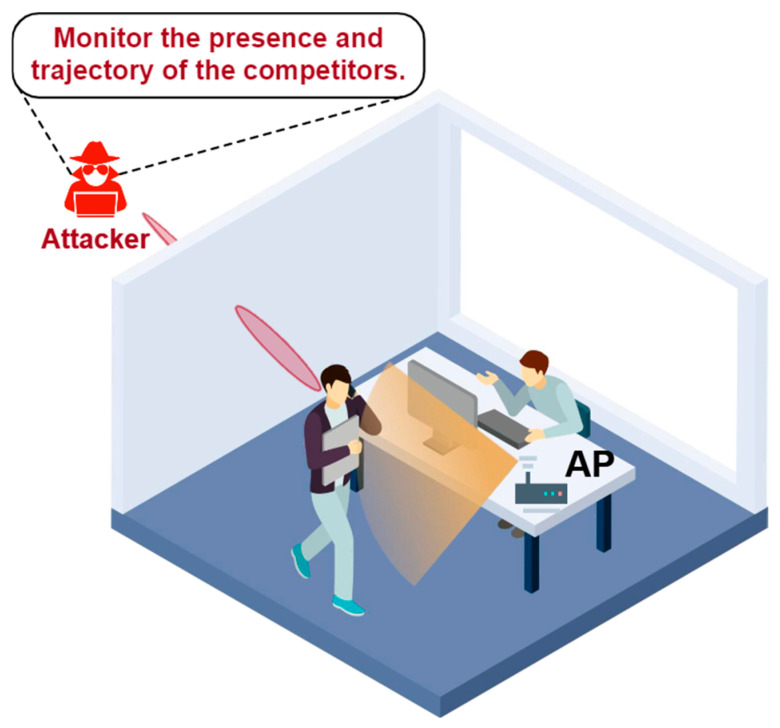
Illustration of the scenario of ActListener [67].

ActListener [67] demonstrates the vulnerability of WiFi sensing where an eavesdropper can eavesdrop on user activities imperceptibly using a WiFi infrastructure in any location of the user sensing area. As shown in Figure 4, this attack can be used to monitor the confidential information of the victims. ActListener can not only estimate the locations of legitimate devices and the victim users but also convert the eavesdropped signals to those of legitimate devices based on the models. Moreover, ActListener is robust to noises in dynamic wireless channels.

WiPeep [52] uncovers loopholes in the 802.11 protocol, which can be exploited to steal the location information of WiFi devices. Specifically, WiPeep first generates fake WiFi traffic from the attack device (e.g., a drone), which will cause the victim device to respond. With this response, WiPeep can use a ToF scheme to locate the victim device even when the device is non-cooperative and in a building.

### 3.3. Discussion

By reviewing the above-mentioned works on the attack techniques against WiFi sensing, some lessons have been learned from these efforts.

**Cost and efficiency**: The cost and efficiency of executing attacks on WiFi sensing systems vary significantly between active and passive approaches, influencing their practicality and prevalence. For active attacks, additional specialized devices are often required to carry out the attack effectively. For example, RIStealth relies on a metasurface to manipulate wireless signals, while WiAdv employs a full-duplex device to interfere with the sensing process. Although these devices can achieve a high attack success rate (e.g., over 70% in both RIStealth and WiAdv), their high cost (e.g., over USD 1000 per device) makes such attacks economically impractical for most attackers. On the other hand, the use of commercial UAVs in attacks like Wi-Peep reduces the cost barrier, with devices priced at around USD 200, making them a more feasible option for attackers. In contrast, passive attacks are far more cost-efficient, as they typically require only a standard WiFi device to receive and analyze wireless signals. This significantly lowers the burden on attackers, both financially and logistically. Moreover, passive attacks often achieve high success rates. For instance, ActListener demonstrates an attack success rate of over 80%, showcasing the effectiveness of this approach. The disparity in cost and efficiency between active and passive attacks highlights a critical consideration for the design of secure WiFi sensing systems. While active attacks may pose a higher technical challenge, their high cost limits their widespread adoption. Passive attacks, on the other hand, are more accessible and equally, if not more, effective, making them a more immediate threat.

**Existing attacks**: It is important to recognize that attacking the wireless channel is generally easier than directly compromising the Tx or Rx devices. This is primarily because the wireless channel serves as an open medium that can be accessed and monitored by anyone within range, making it vulnerable to a wide range of attacks such as signal interference, jamming, or eavesdropping. In contrast, the Tx and Rx devices are typically under the control of authorized users, often equipped with security protocols, authentication mechanisms, and physical access controls that make them much harder to breach. As a result, it is more feasible to exploit the inherent openness of the wireless channel rather than attempting to gain access to APs of the WiFi sensing system.

**Advanced attacks**: Moreover, there are some advanced attack methods exploited in attacking wireless communication systems that may also be used for sensing information eavesdropping in the future. One may better defend against these attacks based on clear knowledge of them. (1) Man-in-the-middle (MITM) attacks [77]: In a MITM attack, the attacker secretly intercepts and potentially alters the communication between two parties without their knowledge. In the context of WiFi sensing, an MITM attacker could position themselves between the sensing device (e.g., a WiFi router) and the target (e.g., a user or object being sensed). By doing so, the attacker could intercept the sensing data, modify them to introduce errors, or even inject malicious data to mislead the sensing system. (2) Supply chain attacks [78]: Supply chain attacks target the hardware, software, or services that are part of the WiFi sensing ecosystem. In such attacks, the attacker compromises a component of the supply chain (e.g., a WiFi chip manufacturer, a firmware provider, or a third-party software library) to introduce vulnerabilities or malicious code into the sensing system. For instance, an attacker could tamper with the firmware of a WiFi router to secretly collect and transmit sensing data to an external server. (3) Proactive eavesdropping: Proactive eavesdropping is increasingly employed to steal communication content [79]. In this type of attack, the attacker actively manipulates the wireless environment to intercept or decode sensitive information rather than passively monitoring the channel. In the context of WiFi sensing, proactive eavesdropping could be adapted to target the sensing process itself. For instance, an attacker might transmit interfering signals to disrupt the sensing system’s ability to accurately detect motion or environmental changes. Alternatively, the attacker could manipulate the wireless channel to extract sensitive information about the sensing target, such as identifying their presence, activities, or even physiological characteristics (e.g., breathing or heart rate).

## 4. Defenses for WiFi Sensing

In this section, we elaborate on the defense techniques for WiFi sensing and against the adversarial attempts of attackers. As summarized in Table 2, we first introduce the related works on active and passive defenses, respectively.

### 4.1. Active Defense

**Figure 5:**
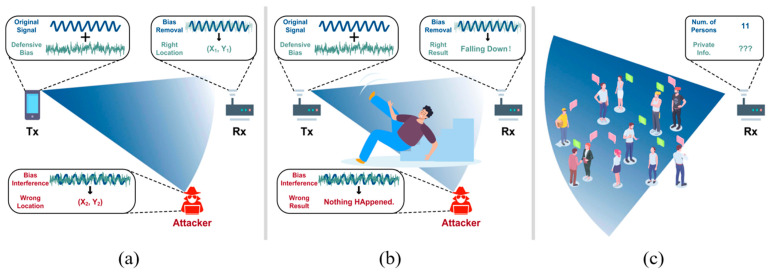
Illustration of defenses for different WiFi sensing applications (e.g., localization, activity recognition, and crowd counting): (**a**) Localization [57]; (**b**) activity recognition [80]; (**c**) crowd monitoring [88].

Active defense refers to proactive measures implemented by the sensing target himself/herself/itself to counteract and mitigate the effects of malicious attacks [56,57,80,81,82,83,84,85]. This strategy involves the deployment of various techniques and mechanisms that enhance the resilience of the sensing system, ensuring integrity and reliability in wireless sensing applications, including localization [57,85], activity recognition [80], and so on, as Figure 5a,b illustrates. The main objective of active defense is to detect and neutralize threats in real time, preventing the leakage of private sensing information. For example, a secure WiFi sensing system equipped with active defense strategies may involve the proactive obfuscation of signals or the use of decoy systems to confuse and misdirect potential attackers. To this extent, active defense strategies can help WiFi sensing systems stay vigilant and adaptive and significantly reduce the risk of successful attacks, ensuring the continued reliability of WiFi sensing applications.

One of the major classes of defense techniques aims to avoid the leakage of the user’s location. MIRAGE [57] involves utilizing the downlink physical layer information as the defense strategy against an attacker snooping on a WiFi user’s location information. Specifically, MIRAGE obfuscates the AoA information of the user’s devices such that the actual direction is along one of the reflected paths, in other words, making the reflected path look like the most direct path from the device to the AP. Thus, the location snooped by the attacker will be significantly deviated from the actual location. It is worth noting that this obfuscation does not affect the communication between WiFi AP and the user and can also be used to defend the ToF-based localization systems.

However, MIRAGE will not only prevent the attackers from obtaining the actual locations but also confuse the legal devices that may provide location-based service to the user. In this case, WiCloak [85] addresses this problem by letting the legal devices know how to recover the deviated location. The basic idea of WiCloak is to manipulate the transmitted WiFi packet to make the calculated CSI at the eavesdropper point useless for localization. To support packet transmission at legitimate receivers, WiCloak also compensates for the CSI change in the payload field and therefore enables standard WiFi decoding on COTS devices. To manipulate the CSI, WiCloak modifies the preamble of WiFi packets and adds an extra fake channel (i.e., extra amplitude and phase) to each symbol. As a result, any Rx will obtain the changed CSI based on the preamble. However, the CSI with this fake channel does not match the actual channel and cannot be used in decoding the payload of WiFi packets, resulting in WiFi communication failure. Thus, WiCloak compensates for the fake channel at Rx by performing reverse operation of manipulation so that the manipulated CSI can be used to decode the payload.

Zhao et al. [82] tried to secure the WiFi localization from the perspective of encryption algorithms. They designed an effective yet lightweight WiFi localization privacy algorithm, which reinforces dummy techniques with plausible dummy locations to resist attacks on the WiFi localization system. The proposed algorithm does not rely on trusted third parties and can resist spatio-temporal correlation attacks.

Another common class of active defense techniques focuses on recognition and detection applications. Zhang et al. [84] first defined the targeted and non-targeted protection of CSI-based WiFi sensing. Although CSI contains a wealth of different semantics, some of which need to be recognized and others that need to be protected. Thus, they constructed an advanced secure WiFi sensing system to exactly modify the private semantic information of CSI while still reserving the other required information. Moreover, to deal with various classifiers, they transformed original signals into adversary signals by training a deep learning architecture with the output of the classifiers. Similarly, Zhou et al. [80] attempted to develop an adversarial deep network architecture for human behavior preservation, which aims to make the targetable private behaviors of a human being not recognizable by general classifiers, while the recognition of other information remains unaffected. To achieve this, the proposed model is capable of intentionally modifying CSI data extracted from received WiFi signals, constraining the new classification results to match the adversarial requirements.

**Figure 6:**
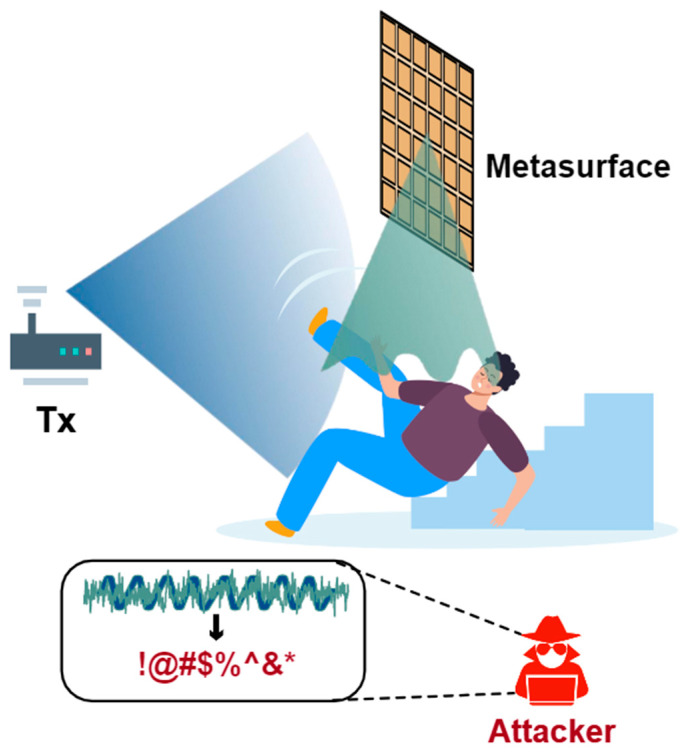
Illustration of the high-level idea of IRShield [56].

To prevent eavesdroppers from obtaining estimations of wireless channels and stealing sensitive information, IRShield [56] was proposed as a novel countermeasure leveraging the advanced capabilities of metasurfaces. As shown in Figure 6, a dedicated algorithm is integrated into the system to generate randomized metasurface configurations, dynamically altering the wireless channel environment to achieve channel obfuscation. This randomness effectively confounds the attacker by providing misleading or confusing sensing results, making it difficult for them to extract accurate information from the wireless signals. As the first practical attempt to exploit metasurfaces as a countermeasure for unauthorized wireless sensing, IRShield represents a significant step forward in privacy-preserving technology. Designed for plug-and-play integration with existing wireless infrastructures, IRShield can be easily deployed as a standalone extension without requiring major overhauls of the current system architecture. This makes it particularly appealing for environments where privacy is paramount.

### 4.2. Passive Defense

Passive defense refers to protective measures adopted by external parties, such as those using the sensing data or third-party entities [58,59,86,87,88]. These defenses operate without requiring direct involvement from the sensing target, focusing on safeguarding the integrity and confidentiality of the sensed information through external mechanisms. By intercepting potential threats or vulnerabilities before they can affect the target, passive defense systems provide an additional layer of security. As the sophistication of wireless attacks continues to grow, passive defense strategies will become increasingly vital in maintaining the privacy and security of wireless sensing systems without relying on direct user intervention.

Focusing on using WiFi sensing for crowd monitoring (e.g., people counting for safety and security management), Rusca et al. [88] also paid attention to the privacy issue. Specifically, a fingerprint-based scheme was proposed to leverage probe request messages emitted by smart devices as a proxy for crowd monitoring. Meanwhile, this solution exploits Bloom filters to ensure formal deniability, aligning with the stringent requirements set forth by regulations like the European GDPR. In that case, this scheme not only addresses the essential task of crowd monitoring but also incorporates advanced privacy-preserving mechanisms, offering a comprehensive approach to managing crowds.

Considering serious privacy concerns raised by cloud-based indoor localization services, Wang et al. [87] proposed a privacy-preserving indoor positioning scheme for WiFi localization based on inner product encryption (IPE) in a cloud environment. They also designed a Bloom filter, which is constructed with locality sensitive hashing, to map WiFi fingerprints from Euclidean to inner product space with the distance relationships maintained for converting the location estimation to inner product calculations. Note that IPE can protect the user’s fingerprint and database information held by the positioning service provider. Fingerprint similarity as determined by the inner product is decrypted on the cloud to retrieve the closest encrypted location coordinates for users. This scheme can effectively ensure sensing security while not significantly degrading the localization accuracy.

Similarly, PriLA [86] also noted the privacy concerns of location authentication with WiFi technologies. Thus, PriLA constructs a privacy-preserving location authentication protocol that facilitates location authentication without compromising users’ location privacy in WiFi networks. Specifically, PriLA leverages carrier frequency offset (CFO) to secure wireless transmission between the mobile user and the AP, meanwhile authenticating the reported locations without leaking the exact location information based on the coarse-grained location proximity being extracted from users’ multipath profiles.

PrivateBus [58] focuses on another novel scenario: public WiFi spots in modern transportation systems (e.g., buses). PrivateBus incorporates two models for uniqueness analyses and sensing information protection. On one hand, a PB-FIND model is utilized to identify the probability that a user can be uniquely re-identified from leaked information, including users’ finger traces (i.e., connection URL and domain), foot traces (i.e., locations), and hybrid traces. On the other hand, a PB-HIDE model protects users from potentially leaked information.

### 4.3. Discussion

By reviewing the above-mentioned works on the defense techniques for WiFi sensing, some lessons can be learned from these efforts.

**Cost and efficiency**: For active defense, users have several options to enhance the security of their WiFi sensing systems, including employing additional devices or modifying the WiFi hardware or firmware. For instance, IRShield leverages a metasurface to reduce the detection rate of attackers to less than 5%. However, this approach comes with a significant cost, as dedicated metasurfaces can exceed USD 1000, making it a less feasible option for many users. Another example is WiCloak, which modifies the WiFi hardware to increase the localization error by 22 times compared to standard WiFi systems. While this method is effective, it requires technical expertise to implement, which may be beyond the capabilities of typical users. For normal users without specialized knowledge, purchasing an additional device is often the most practical choice. However, the high cost of such devices can be a deterrent. In this case, turning to a third-party solution for protection at a moderate cost may be a more viable alternative, in other words, choosing passive defense. Third-party solutions can provide advanced security features without requiring users to modify their hardware or firmware directly. This approach not only reduces the burden on users but also ensures that the system remains secure against evolving threats.

**Sensing target**: Existing defense techniques are designed to protect the wireless channel from unauthorized access, ensuring that sensitive data are shielded from potential attackers. However, some of these strategies focus on obfuscating the wireless channel entirely to prevent attackers from extracting useful information. While this approach successfully blocks adversaries from accessing the channel, it presents a significant drawback: legitimate users are also unable to perform sensing operations or provide related services. This compromise essentially limits the core functionality of the system, raising concerns about its practical deployment, especially in environments where continuous and reliable sensing is critical. Moreover, several defense mechanisms propose altering the hardware or software of commercial devices or modifying the format of WiFi packets to enhance security. While these techniques may offer robust protection in theory, they come with notable limitations in real-world applications. The requirement to modify commercial off-the-shelf devices is often impractical, as most users and organizations lack the resources, technical expertise, or willingness to implement such changes. Additionally, altering standard protocols or packet structures can introduce compatibility issues, making it difficult to integrate these defenses into existing networks. These constraints significantly hinder the availability and scalability of these methods, limiting their usefulness in large-scale or widely distributed systems where uniform adoption is challenging.

**Third parties**: While some efforts have been made to secure WiFi sensing through the involvement of external entities, this introduces additional concerns regarding the trustworthiness and security of these third parties. Relying on external organizations or service providers to manage or secure sensing operations raises the question of whether these parties can be fully trusted to handle sensitive data responsibly and without introducing new vulnerabilities. The involvement of third parties inevitably expands the attack surface, as it creates more points of potential failure or compromise. If these external entities are not properly vetted or secured, they themselves could become targets for attackers, ultimately undermining the very security they are meant to uphold. To mitigate these risks, cryptographic algorithms can be employed to enhance the security of WiFi sensing when third parties are involved.

## 5. Challenges and Opportunities

According to the above discussion, we can see that there is still a big gap between the state-of-the-art technologies and the ideal vision of secure WiFi sensing. Filling this gap requires continuous innovations in all the dimensions around this technology, involving the efforts of WiFi sensing algorithm developers and even attackers. Therefore, our discussion in this section accordingly focuses on these two main aspects to point out the potential challenges and opportunities in future research and deployment of secure WiFi sensing systems.

### 5.1. Attackers

**(1) Imperceptible Adversarial Attack**: Even though many existing attack techniques can effectively pose significant threats to WiFi sensing systems, they still have large vulnerabilities that could be detected or mitigated by defensive mechanisms. For attackers, one major challenge is to develop imperceptible adversarial attack techniques that can manipulate WiFi signals in real time without being noticed by the system or triggering alarms. This demands a deep understanding of signal processing, as WiFi sensing systems rely heavily on the fine-grained manipulation of CSI and RSSI. Attackers must also be well versed in wireless communication protocols to manipulate signal characteristics at a low level and also in adversarial machine learning, which can help in generating subtle perturbations or modifications to signals that evade detection.

The opportunity for attackers lies in exploiting vulnerabilities in the algorithm’s processing pipeline—especially in how these systems interpret and classify changes in the wireless environment. By understanding how these systems process data from different antennae, frequencies, and channels, attackers can develop techniques that introduce imperceptible alterations to the signal flow. These manipulations could lead to incorrect sensing results, such as generating ghost objects, altering object trajectories, or misidentifying activities, all without the system realizing that it has been compromised, which could raise the attack success rate.

Ultimately, the development of such advanced techniques could expose critical security gaps in current WiFi sensing systems, paving the way for future advancements in defensive mechanisms. The study of these vulnerabilities could inspire algorithm developers to design more robust systems that incorporate adversarial training or real-time anomaly detection to defend against such sophisticated attacks. By identifying and addressing these weaknesses early, the field can move towards creating more secure and resilient WiFi sensing technologies.

**(2) Advanced Attack against Defensive Means**: There are already many corresponding defense measures for common attack techniques targeting WiFi sensing systems, including encryption, signal obfuscation, jamming detection, and anomaly detection algorithms. These measures are designed to safeguard the integrity and accuracy of WiFi sensing, protecting against threats like spoofing, jamming, and adversarial attacks. However, attackers are constantly evolving their methods, striving to bypass these defenses and exploit system vulnerabilities. To deal with these existing security measures, attackers must develop more advanced, stealthy, and adaptive attack techniques that can evade detection and overcome countermeasures.

For instance, traditional spoofing attacks involve injecting falsified data into the WiFi sensing system to generate incorrect outputs. However, these attacks often create inconsistencies that can be detected by anomaly detection mechanisms. To bypass these defenses, attackers can develop context-aware spoofing attacks, where they gather detailed environmental data in real time and use this information to craft more realistic and undetectable spoofing signals. If a WiFi sensing system is being used to monitor human movements in a room, an attacker could carefully observe the movements of individuals over time and adjust their spoofing attacks to match the expected patterns. By aligning the spoofed signals with real-world events, attackers can make it much harder for the system to distinguish between legitimate and falsified data. This kind of context-aware attack would require sophisticated sensing and machine learning techniques to model the environment and make dynamic adjustments to the spoofed signals.

To further deal with defense measures, attackers can combine multiple attack vectors into coordinated multi-modal attacks, where different aspects of the WiFi sensing system are targeted simultaneously. For instance, an attacker might simultaneously launch a spoofing attack while also introducing jamming or timing manipulation. By attacking from multiple angles, attackers can overwhelm the system’s defenses, which are often designed to handle one type of attack at a time. These multi-modal attacks could exploit the interactions between different parts of the system to create complex failure modes that are difficult for the system to isolate or mitigate. For example, by jamming certain frequencies while introducing subtle spoofing signals on another, attackers can confuse the system’s decision-making process and evade detection. The challenge here is for attackers to develop a deep understanding of the interdependencies between various WiFi sensing components and defense mechanisms to launch successful, highly targeted attacks.

These advanced attacks can catalyze significant advancements in both WiFi sensing and defense technologies. The continuous interplay between attack and defense not only strengthens the security of WiFi sensing systems but also fosters innovation in the field, leading to more effective and adaptive sensing technologies.

### 5.2. WiFi Sensing Algorithm Developers

**(1) Reducing Impact on WiFi Communication Systems**: Many existing defense methods aimed at protecting WiFi communication systems can significantly impact their performance, often leading to hindered data communication and diminished functionality of WiFi devices. For instance, they may introduce latency, reduce data throughput, and increase computational overhead, which in turn potentially compromises the overall efficiency and user experience of WiFi communication networks.

In light of these challenges, future developments in secure WiFi sensing algorithms must focus on mitigating the impact of these defenses while preserving the integrity and performance of WiFi communication. This involves developing advanced algorithms that integrate seamlessly with existing communication protocols without introducing significant overhead or latency. One approach could be to design adaptive security mechanisms that dynamically adjust their level of protection based on the context and the perceived threat level, thereby balancing security and performance. For example, algorithms could implement context-aware encryption that adapts the encryption strength based on the sensitivity of the transmitted data and the security requirements of the specific application.

On one hand, innovations in efficient signal processing and machine learning can lead to more effective defenses that have minimal impact on WiFi communication. Techniques such as lightweight cryptographic algorithms and real-time anomaly detection can help reduce computational demands and maintain high throughput. The use of edge computing can also offload some of the processing tasks from the WiFi devices themselves to edge servers, further minimizing the performance impact. On the other hand, future research could explore novel approaches to integrating security and communication more holistically. This might include developing secure communication protocols that inherently incorporate robust defense mechanisms without compromising efficiency. For instance, protocols designed with built-in resilience to attacks could prevent the need for separate security layers that disrupt communication.

**(2) Privacy Preservation in Secure WiFi Sensing**: Privacy is a significant concern in WiFi sensing applications and systems, particularly when these systems are deployed in sensitive environments such as homes and offices. These environments are often rich with personal and confidential information, and any compromise in privacy can have serious implications for individuals and organizations. Therefore, developers face the challenge of creating algorithms that not only protect user data but also maintain the functionality and accuracy of the sensing system.

One effective approach to addressing privacy concerns is the use of differential privacy techniques [89]. Differential privacy involves adding noise to the data collected by the system to obfuscate specific details, ensuring that individual data points cannot be identified or reconstructed. This technique provides a mathematical guarantee that the inclusion or exclusion of a single data point will not significantly affect the outcome of the analysis, thus protecting user privacy. Another promising avenue is secure multiparty computation (SMC) [90]. SMC allows multiple parties to jointly compute a function over their combined data without revealing their individual inputs to any other party. In the context of WiFi sensing, this means that data from different sources can be aggregated and processed to derive useful insights without exposing sensitive information to any single entity.

Furthermore, developers should consider the use of privacy-by-design principles in the development process. This approach involves embedding privacy protections into the system architecture from the outset, ensuring that privacy considerations are integrated into every aspect of the system’s design and operation. By adopting privacy-by-design principles, developers can proactively address potential privacy issues and build systems that are resilient to emerging threats.

## 6. Conclusions

Secure WiFi sensing has gained significant attention as WiFi sensing technologies become increasingly integrated into critical domains such as smart homes, medical health monitoring, and security monitoring. With the growing reliance on these applications, the demand for privacy and security has become more pressing than ever. In this survey, we have provided a comprehensive overview of recent research advances in secure WiFi sensing, focusing on both attack and defense technologies. Our work offers a complete picture of the field, introducing a novel taxonomy for classifying existing works into active and passive approaches. This taxonomy provides a structured framework for understanding the evolving threat landscape and defense strategies in WiFi sensing. Additionally, we have presented an inclusive and up-to-date coverage of the literature, thoroughly comparing and summarizing the latest advancements in the field. By identifying potential new attack methods and emerging defense techniques, we aimed to highlight the critical challenges and opportunities in secure WiFi sensing. We hope that this survey not only brings attention to this important topic but also inspires further research and innovation in both industry and academia.

## Figures and Tables

**Table 1:** Comparison of attack techniques.

Method	Category	Attack Aim	Sensing Scenario	Potential Defense
WiAdv [60]	Active attack	Introducing disturbance to the channel to decrease the sensing performance	Gesture recognition	Physical isolation Interference suppression
Liu et al. [61]	Active attack	Introducing CSI absence to decrease the sensing performance	Gesture recognition	Physical isolation Interference suppression Anomaly detection
Song et al. [62]	Active attack	Injecting false data to sensing datasets to decrease the sensing performance	Activity recognition	Adversarial training Anomaly detection
Li et al. [51]	Active attack	Manipulating WiFi preambles to decrease the sensing performance	Activity recognition User authentication	Adversarial training Anomaly detection
Liu et al. [63]	Active attack	Introducing disturbance to the channel to decrease the sensing performance	Location estimation	Adversarial training Anomaly detection
WiCAM [64]	Active attack	Introducing adversarial noise in the received signals to decrease the sensing performance	Gesture recognition Activity recognition User authentication	Adversarial training Anomaly detection
RIStealth [65]	Active attack	Rendering a moving individual undetectable by WiFi intrusion detection systems	Intrusion detection	Anomaly detection Device upgrade
IS-WARS [54]	Active attack	Injecting cross-technology signals to the channel to decrease the sensing performance	Activity recognition	Signal processing Adversarial training
Shi et al. [66]	Passive attack	Identifying users’ private information in sensing datasets, like height and weight	Gesture recognition Activity recognition	Data anonymization
Hernandez et al. [55]	Passive attack	Tracking and counting the flow of traffic throughout a building	Occupancy monitoring Crowd counting	Physical isolation Channel disturbance
ActListener [67]	Passive attack	Eavesdropping on user activities imperceptibly in any location of user sensing area	Activity recognition	Physical isolation Channel disturbance
WiPeep [52]	Passive attack	Eavesdropping locations of indoor WiFi devices	Location estimation	Randomizing ToF Fake packets detection

**Table 2:** Comparison of defense techniques.

Method	Category	Defense Aim	Sensing Scenario	Targeted Attack
Zhou et al. [80]	Active defense	Misclassifying the private type of information while the others still are recognizable	Activity recognition	Information eavesdropping Behavioral snooping
Wobly [81]	Active defense	Anonymizing users’ identities when performing accurate gait authentication	Gait recognition	Information eavesdropping Behavioral snooping
IRShield [56]	Active defense	Obfuscating wireless channels to prevent overhearing of sensitive information	Motion detection	Information eavesdropping Behavioral snooping
MIRAGE [57]	Active defense	Utilizing the downlink physical layer information to defense eavesdropper	Location estimation	Information eavesdropping Location snooping
Zhao et al. [82]	Active defense	Creating plausible dummy locations to preserve indoor localization information	Location estimation	Information eavesdropping Location snooping
SecureSense [83]	Active defense	Achieving consistent predictions of WiFi sensing systems regardless of input perturbations	Activity recognition	Adversarial attack
Zhang et al. [84]	Active defense	Decreasing the recognition accuracy of the protected semantic significantly	User authentication Activity recognition	Information eavesdropping Identity snooping Activity snooping
WiCloak [85]	Active defense	Rendering location information obtained by eavesdroppers meaningless	Location estimation	Information eavesdropping Location snooping
PriFi [59]	Passive defense	Helping users to perceive ongoing observations from systems in the user’s current environment	\	Information eavesdropping
PriLa [86]	Passive defense	Facilitating location authentication without compromising user’s location privacy	Location estimation	Information eavesdropping Location snooping
Wang et al. [87]	Passive defense	Protecting user’s positioning results in cloud-based indoor positioning systems	Location estimation	Information eavesdropping Location snooping
Rusca et al. [88]	Passive defense	Performing crowd counting and tracking within large-scale gatherings	Crowd monitoring	Information eavesdropping Crowd flow snooping
PrivateBus [58]	Passive defense	Protecting users from the leaked information in bus WiFi systems	Foot trace tracking Finger trace tracking	Information eavesdropping Trace snooping

## Data Availability

Data are subject to third-party restrictions.
